# Supplementary material for: Acquisition and Exaptation of Endogenous Retroviruses in Mammalian Placenta
Source: Biomolecules. 2023 Oct 4;13(10):1482. doi: 10.3390/biom13101482 (PMC10604696; doi:10.3390/biom13101482)
Supplement: Supplementary file 1 [file biomolecules-13-01482-s001.zip › biomolecules-2585581-supplementary.pdf]

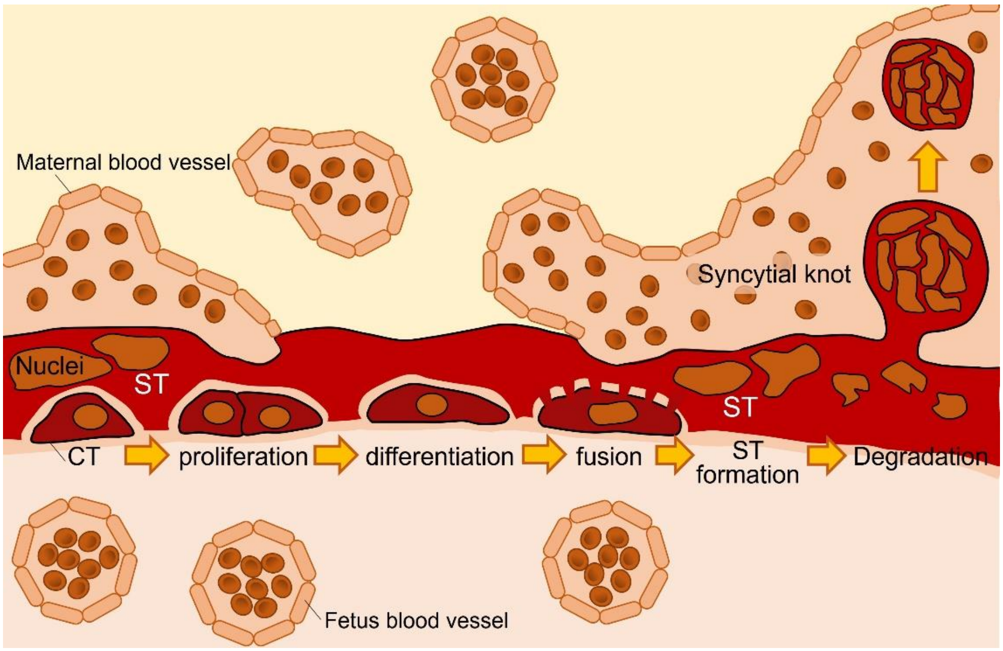

Figure S1. Schematic representation of trophoblast differentiation.

Cytotrophoblasts differentiate and increase their number of intracellular organelles, mRNA, and proteins. Differentiated cytotrophoblasts fuse with overlying syncytiotrophoblasts. Eventually, the intracellular organelles are degraded, and the nuclei of late apoptotic syncytiotrophoblasts accumulate in the syncytial knots and are released into the maternal bloodstream. Arrows indicate the routes of cellular changes during trophoblast differentiation. CT, Cytotrophoblast; ST, Syncytiotrophoblast.
